# RETRACTION: Efficacy and Safety of Ultrasound‐Guided Foam Sclerotherapy Combined With Endoluminal Radiofrequency Closure in Patients With Varicose Veins of Lower Extremities

**DOI:** 10.1111/iwj.70560

**Published:** 2025-04-18

## Abstract

**RETRACTION:** ZhaoC. , LiD. , and XieC. , “Efficacy and Safety of Ultrasound‐Guided Foam Sclerotherapy Combined With Endoluminal Radiofrequency Closure in Patients With Varicose Veins of Lower Extremities,” International Wound Journal 20, no. 7 (2023): 2518–2527, 10.1111/iwj.14116.36796354 PMC10410358

The above article, published online on 16 February 2023, in Wiley Online Library (http://onlinelibrary.wiley.com/), has been retracted by agreement between the journal Editor in Chief, Professor Keith Harding; and John Wiley & Sons Ltd. Following an investigation by the publisher, all parties have concluded that this article was accepted solely on the basis of a compromised peer review process. The editors have therefore decided to retract the article. The authors did not respond to our notice regarding the retraction.

**RETRACTION:** C. Zhao , D. Li , and C. Xie , “Efficacy and Safety of Ultrasound‐Guided Foam Sclerotherapy Combined With Endoluminal Radiofrequency Closure in Patients With Varicose Veins of Lower Extremities,” International Wound Journal 20, no. 7 (2023): 2518–2527, 10.1111/iwj.14116.36796354 PMC10410358

The above article, published online on 16 February 2023, in Wiley Online Library (http://onlinelibrary.wiley.com/), has been retracted by agreement between the journal Editor in Chief, Professor Keith Harding; and John Wiley & Sons Ltd. Following an investigation by the publisher, all parties have concluded that this article was accepted solely on the basis of a compromised peer review process. The editors have therefore decided to retract the article. The authors did not respond to our notice regarding the retraction.

